# Neurological Resilience in Patients With Anorexia Nervosa Following Prolonged Cardiopulmonary Resuscitation

**DOI:** 10.7759/cureus.55429

**Published:** 2024-03-03

**Authors:** Shigetoshi Ogiwara, Soichiro Wada, Shuji Sai, Takuya Tamura

**Affiliations:** 1 Department of Pediatric Critical Care, Teine Keijinkai Hospital, Sapporo, JPN; 2 Department of Pediatrics, Teine Keijinkai Hospital, Sapporo, JPN

**Keywords:** extracorporeal cardiopulmonary resuscitation, extracorporeal membrane oxygenation, cardiopulmonary resuscitation, cardiac arrest, anorexia nervosa

## Abstract

Anorexia nervosa (AN) is a psychiatric disorder with metabolic abnormalities. Prolonged cardiopulmonary resuscitation (CPR) is predicted to result in death and poor neurological outcomes. This report describes the case of a patient with AN who had an unexpectedly favorable outcome after prolonged CPR. A 12-year-old female with AN presented to the emergency department, requiring intubation due to worsening consciousness and respiratory distress. Refractory hypotension led to cardiac arrest. After 135 minutes of CPR, venoarterial extracorporeal membrane oxygenation (EMCO) was started, and the patient was treated for post-resuscitation management, refeeding syndrome, and sepsis. The cardiac function gradually improved, the patient was weaned from EMCO eight days after admission, and the patient was extubated 30 days after admission. The patient maintained a good central nervous system function. AN patients tend to be youngsters and have a lower metabolism, which may be associated with a favorable neurological prognosis after prolonged CPR.

## Introduction

In general, survival rates are inversely related to the increasing duration of cardiopulmonary resuscitation (CPR) for any reason. Prolonged CPR is predicted to result in resuscitation failure or severe neurological prognosis [1]. It has been reported that neurological prognosis is relatively preserved after prolonged resuscitation with extracorporeal cardiopulmonary resuscitation (ECPR) compared to conventional CPR [2].

Anorexia nervosa (AN) is a life-threatening psychiatric disorder that can cause sudden and unexpected cardiac arrest. The long-term mortality from AN-related diseases has been reported to be 15% [3]. Half of the cardiac arrests in AN cases have been reported to be due to suicide, whereas the majority of the remaining cases are associated with fatal arrhythmias and refeeding syndromes [4]. However, there are only a few reports of patients with AN who have been resuscitated after cardiac arrest [5,6].

This report describes a case of a pediatric female patient with anorexia nervosa who was transferred to a tertiary care facility due to cardiac arrest secondary to sepsis and refeeding syndrome and had a favorable outcome after ECPR and conventional CPR.

## Case presentation

A 12-year-old female with AN visited the ED of a regional hospital (secondary care hospital) due to fever and malaise. She had already been diagnosed with AN according to the Diagnostic and Statistical Manual of Mental Disorders, Fifth Edition, (DSM-5) criteria by a child psychiatrist two months prior to admission. At the time of presentation, she had lost 12.7 kg since six months earlier (body weight 27.4 kg; height: 157.0 cm; BMI: 11.1). Vital signs at the time of presentation were a heart rate of 90 bpm, systolic blood pressure of 120 mmHg, SpO2 of 95%, and body temperature of 40°C. She was severely emaciated and dehydrated. Blood counts and biochemistry measurements were within normal limits, except for hyperglycemia and mild abnormal thyroid function (potassium 3.9 mEq/L, phosphorus 4.6 mg/dl, blood glucose 323 mg/dl, thyroid stimulating hormone (TSH) 0.47 μU/ml (0.50-5.0), free triiodothyronine (T3) 1.57 pg/ml (2.3-4.0), free thyroxine (T4) 0.81 ng/dl (0.90-1.70)).

Extracellular fluid replacement therapy, including glucose and vitamin B, was started in the ED. However, several hours after the onset of treatment, she suddenly developed impaired consciousness accompanied by severe respiratory distress that required intubation. At that time, the serum phosphorus concentration had dropped to 2.1 mg/dl. Emergent pediatric advanced life support was initiated because she developed refractory shock status, eventually leading to cardiac arrest. The initial rhythm was pulseless electrical activity. After 60 minutes, spontaneous circulation returned, and she was transported to our hospital (tertiary care hospital) for further intensive therapy. During transport, she had another cardiac arrest, and CPR was resumed. The patient was difficult to ventilate due to a large amount of blood-tinged frothy sputum. Upon arrival at the hospital, extracorporeal membrane oxygenation (EMCO) was immediately started after a total of 135 minutes of CPR.

**Figure 1:**
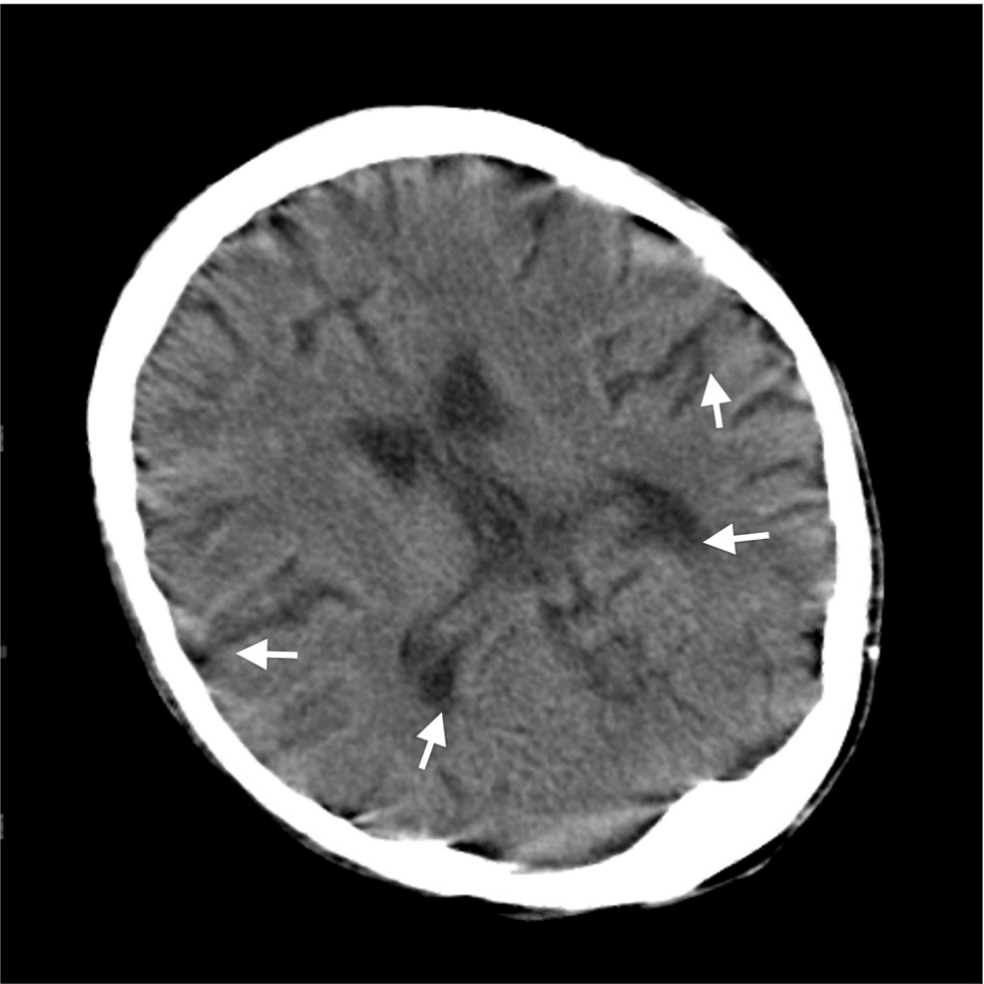
Brain CT on admission showed mild brain atrophy, suspected to be caused by long-term malnutrition

**Figure 2:**
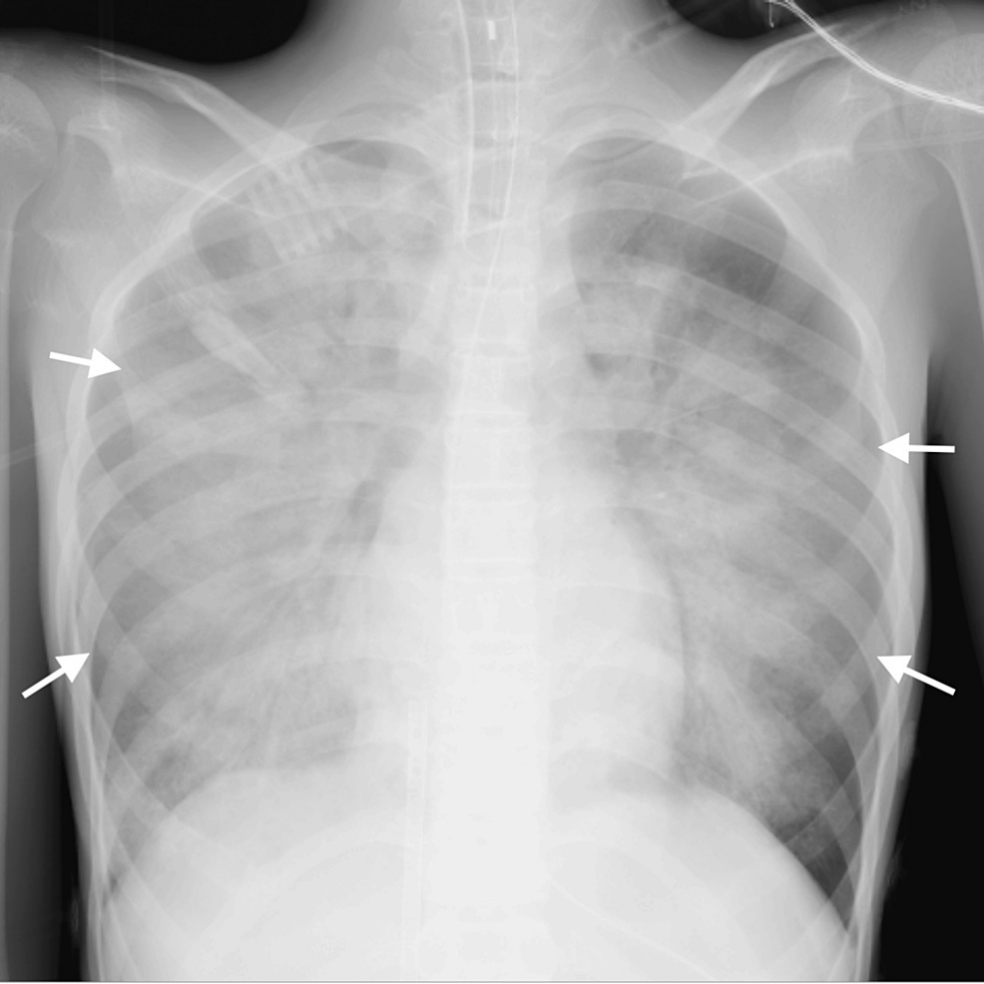
Chest radiography showed extensive bilateral pulmonary edema on admission

The cardiac arrest could be attributed to refeeding syndrome and sepsis. She was admitted to the ICU where careful electrolyte management and antibiotic therapy were initiated. The head CT and chest X-ray on admission to the ICU are shown in Figure 1 and Figure 2, respectively. On the next day, Escherichia coli (E. coli) was detected in a blood culture. The cardiac function gradually recovered; she was successfully weaned from ECMO eight days after admission. Long-term mechanical ventilation was required due to severe muscle weakness, but after recovery, extubation was successfully performed 30 days after admission. Her memory and cognitive functions were fully preserved, although she continued to receive medical treatment for chronic heart failure and gait rehabilitation for the complications of left peroneal nerve palsy. Four months after admission, she was transferred to the psychiatric ward.

## Discussion

The most notable feature of this case was the extremely long resuscitation time. Favorable outcomes in AN patients with shorter resuscitation times have been reported previously where a 22-year-old patient with AN with ventricular fibrillation due to refeeding syndrome was saved after 45 minutes of CPR followed by ECMO. Her cardiac function recovered with no neurological sequelae [5]. In another report, a 33-year-old patient with AN had a cardiac arrest due to left ventricular dysfunction during hospitalization and recovered after immediate ECMO [6]. In comparison to the cases, the patient in this study had an even longer duration of CPR (Table 1) [5-8].

**Table 1:** A summary of the reported cases of sudden resuscitation events in patients with anorexia

Author, year	Age, sex	Cause of cardiac arrest	Low-flow times	ECMO	Prognosis	References
Bonnemeier, et al. 2006	22-year-old, female	Catecholamine cardiomyopathy	Unknown	−	Death	[7]
Ono, et al. 2009	33-year-old, female	Cardiac dysfunction	Unknown	+	Neurologically favorable survival	[6]
Ewan, et al. 2013	16-year-old, female	Arrhythmia with hypokalemia	Unknown	−	Neurologically favorable survival	[8]
Waddell, et al. 2020	22-year-old, female	Refeeding syndrome	45 minutes	+	Neurologically favorable survival	[5]
Present case	13-year-old, female	refeeding syndrome and sepsis	135 minutes	+	Neurologically favorable survival	−

Herein, the neurological prognosis was unexpectedly favorable despite the prolonged cardiopulmonary arrest, a common poor prognostic factor. The mortality rate of ECPR for in-hospital cardiac arrest in children has been reported to be 59% [9]. Initial non-shockable rhythm, non-cardiac disease, organ failure prior to ECMO, and long resuscitation time are considered additional poor prognostic factors [9]. In general, conventional CPR for longer than 30 minutes can cause severe neurological and fatal outcomes, but on which patients prolonged resuscitation should be performed has not been determined.

Accidental hypothermia is a critical factor for favorable recovery from prolonged cardiac arrest, where the decreased oxygen consumption associated with hypothermia is believed to protect the brain and organs. Conversely, it has been reported that patients with AN have a reduced metabolism to conserve energy under prolonged hyponutrition, resulting in a significant decrease in core body temperature. [10]. It has also been reported that oxygen consumption is reduced by as much as half in the cells of patients with AN due to mitochondrial dysfunction [11]. Therefore, patients with AN may have resilience to prolonged CPR. In the present case, a decreased serum thyroid hormone level (the pattern of non-thyroidal illness syndrome), which suggests hypometabolism and hypothermia (33.5°C), was also observed at the time of ICU admission. These findings may partially contribute to resilience to cardiopulmonary resuscitation, although our hypothesis requires confirmation in a larger number of cases.

Other common favorable prognostic factors for resuscitation include being a younger patient, bystander CPR, and in-hospital onset. ECPR is reported to have a better long-term prognosis than conventional CPR [2]. A report from the Extracorporeal Life Support Organization has indicated that ECPR in children resulted in a better prognosis than in adults [12]. This is because the ECPR in children occurred mainly in hospitals and the duration of cardiac arrest was short, and cervical or central vessels were used.

## Conclusions

The peak incidence of AN occurs during the teenage years, and AN patients have been reported to exhibit a low basal metabolism. This may be associated with a favorable neurological prognosis after prolonged CPR. Despite insufficient evidence, aggressive ECMO may be considered in young patients with AN who have experienced prolonged cardiac arrest, as in this case, because of the favorable outcome in some cases. However, further rigorous studies with larger sample sizes and appropriate controls are needed to confirm or refute these findings. The use of ECMO in emergency medicine requires careful prognosis estimation due to the crucial resources it demands. Despite this, data predicting the efficacy of ECMO are limited. Emergency physicians should be aware that patients with severe AN are at risk of cardiac arrest. Because pediatric ECMO and quality postresuscitation care require a specialist, emergency physicians should consider early transport to a facility that can provide pediatric ECMO in these cases.
